# Supramolecular Cyclodextrin-Based Hydrogels for Controlled Gene Delivery

**DOI:** 10.3390/polym11030514

**Published:** 2019-03-19

**Authors:** Ana Rey-Rico, Magali Cucchiarini

**Affiliations:** 1 Cell Therapy and Regenerative Medicine Unit, Centro de Investigacións Científicas Avanzadas (CICA), Universidade da Coruña, Campus de A Coruña, 15071 A Coruña, Spain; 2 Center of Experimental Orthopaedics, Saarland University Medical Center, D-66421 Homburg/Saar, Germany; mmcucchiarini@hotmail.com

**Keywords:** supramolecular hydrogels, cyclodextrin-based polypseudorotaxane hydrogels, controlled gene delivery, nonviral vectors, viral vectors, gene transfer

## Abstract

Controlled delivery of gene transfer vectors is a powerful strategy to enhance the temporal and spatial presentation of therapeutic agents in a defined target. Hydrogels are adapted biomaterials for gene delivery capable of acting as a localized depot of genes while maintaining the long term local availability of DNA vectors at a specific location. Supramolecular hydrogels based on cyclodextrins (CDs) have attracted considerable attention as potential biomaterials in a broad range of drug delivery applications. Their unique characteristics of thixotropicity and low cytotoxicity due to their production under mild conditions make them potential candidates to form injectable delivery systems. This work aims to provide an overview of the use of CD-based polypseudorotaxane hydrogels as controlled gene delivery systems for different applications in regenerative medicine.

## 1. Introduction

### 1.1. Cyclodextrins

**Figure 1:**
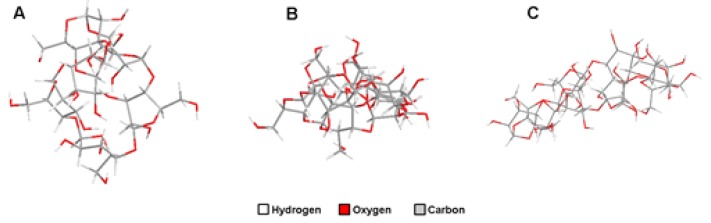
Structure of cyclodextrins. Two-dimensional sequences of α-CDs (CID: 444913) (**A**), β-CDs (CID: 444041) (**B**), and γ-CDs (CID: 5287407) (**C**) were obtained from PubChem Compound. Three-dimensional (3D) structures of the different compounds were drawn with ChemBioOffice 2012 (Chem3D Pro 13; PerkinElmer Informatics).

Cyclodextrins (CDs) represent a group of cyclic oligosaccharides consisting of a relatively hydrophobic cavity and a hydrophilic external face obtained from enzymatic transformation of starch with a torus-like molecular shape [1]. CDs are classified as α-, β, or γ-CDs, depending on the number of D(+)-glucose units linked by α-1,4-linkages, being of 6, 7, and 8, respectively [2] (Figure 1). Moreover, the presence of 2-, 3-, and 6-hydroxyl groups on the ring makes CDs versatile platforms for structural modifications to improve their natural properties in different applications [3]. In addition, CDs are generally considered as safe (GRAS) substances by the Food and Drug Administration (FDA) [1].

CDs have the ability to thread along certain polymer regions (main-chain complexes) or lateral chains (side-chain complexes), leading to the formation of supramolecular assembled structures. These supramolecular structures are normally referred to as polypseudorotaxane when CDs can reversibly travel along the polymer backbone or lateral chains. Conversely, when both ends of the polymer chains in polypseudorotaxanes are covalently capped with bulky molecules, CDs are entrapped and cannot be de-threaded from the assembly, giving so-called polyrotaxanes [4,5].

### 1.2. CD-Based Polypseudorotaxane Hydrogels

Supramolecular hydrogels represent a type of biomaterial consisting of a solid three-dimensional network formed *via* noncovalent bonds, such as a hydrogen bond, hydrophobic interaction, and cation–π and π–π interactions [6].

CD-based polypseudorotaxanes generally consist of polymers, such as poly(ethylene oxide) (PEO) or poly(ethylene glycol) (PEG) [7], poly(propylene oxide) (PPO) [8], or copolymers having blocks of PEO and/or PPO, alone as PEO-PPO-PEO (Pluronics) [9,10,11], or combined with other blocks, such as poly[(R)-3-hydroxybutyrate] [12] or poly(caprolactone) (PCL) [13,14,15].

Supramolecular hydrogels based on polypseudorotaxanes have already been reported as potential candidates for different tissue engineering applications due to their thixotropic nature and excellent biocompatibility, showing vast potential as injectable hydrogel carriers that may be administered *via* non-invasive implantation [16]. Likewise, the mild conditions in the absence of organic solvents under which polypseudorotaxane gels are formed allow for the incorporation of a variety of biomolecules, also encompassing hydrophobic molecules that benefit from the presence of free CDs or micelle-like structures for drug hosting [1,16,17].

## 2. Gene Transfer Vectors: Basic Concepts

### 2.1. Nonviral Vectors

Gene transfer *via* nonviral vectors (transfection) is based on the incorporation of DNA, either naked but mostly complexed with cationic polymers or with cationic lipids (in polyplexes and lipoplexes) into a target population [18]. As a result of this complexation, DNA cargo may be protected against degradation by nucleases and serum components by creating a less negative surface charge [19]. Still, and unlike viral counterparts that have evolved to overcome cellular and immune defense mechanisms, nonviral carriers exhibit reduced transfection efficiencies as they are precluded by numerous extra- and intracellular obstacles [18]. Therefore, the obtaining of an efficient nonviral-mediated transfer might need repeated administration to achieve satisfactory gene therapeutic effects, due to their short retention time or low therapeutic efficacy in target tissues [15]. However, the main advantage of these types of vectors is their biosafety as they avoid the risk of acquiring replication competence and of insertional mutagenesis commonly associated with viral vectors. Likewise, its potential for large scale production at relatively low expense makes these vectors attractive tools for gene therapy [20].

### 2.2. Viral Vectors

Viral gene transfer (transduction) is based on the natural cellular entry pathways of viruses from which they are derived [20]. The most common viruses manipulated for gene transfer purposes include herpes simplex virus (HSV) [21,22], adenoviruses [23,24], retro- and lentiviruses [25,26,27], and adeno-associated virus (AAV) [28,29,30]. While gene transfer *via* viral vectors is highly efficient, the existence of patient-associated factors and physiological barriers (high immunogenicity, inhibition of transduction in the presence of specific anticoagulants) may hinder the effective delivery, processing, and expression of transgenes in the target cells [31,32,33].

## 3. Controlled Delivery of Gene Transfer Vectors *via* CD Hydrogels

### 3.1. Principles of Controlled Gene Delivery

The controlled delivery of gene transfer vectors represents a powerful tool to address the issues associated with the use of gene transfer vectors in clinical settings (i.e., reduced efficiency, rapid degradation, physiological barriers, and/or vector- and patient-specific- immune responses) [33]. In addition, sustained gene expression has been reported to be more effective compared with the administration of recombinant molecules [34], providing high levels of therapeutic genes to target various physiological mechanisms, such as angiogenesis [35] or chondrogenesis [11], to durably enhance the repair of injured tissues and organs [36]. Hereof, the prolonged presence of the gene transfer vectors in the cellular microenvironment may improve the efficacy of the therapeutic cargo by providing a long term, sustained at a specific place treatment while minimizing the exposition of non-target tissues [15,34,37].

Among a variety of biomaterials, hydrogels have been reported as potential tools for gene delivery, affording both protection of the gene transfer vectors against extracellular degradation and premature aggregation and controlled supply of the therapeutic molecules at the target place.

### 3.2. Controlled Delivery of Gene Transfer Vectors via Supramolecular-Based CD Hydrogels

While polypseudorotaxane hydrogels have been extensively studied for drug delivery approaches [1,4], their application in gene delivery approaches has been less explored. In this regard, most applications using CDs for gene therapy purposes focused on the use of polyrotaxanes based on CD-containing cationic polymers acting as carriers of plasmid DNA.

Gene delivery from polypseudorotaxanes hydrogels has been chiefly associated with the shedding of CDs from the linear polymer backbone, being the de-threading rate proportional to the volume of the dissolution medium [38]. Therefore, when injected into the body, polypseudorotaxane hydrogels might gradually dilute upon contact with physiological fluids leading to CD fall-off and the released genes being absorbed by surrounding cells through endocytosis [38].

Here, we will explore the use of polypseudorotaxane hydrogels as controlled delivery systems for gene transfer vectors and their potential application in tissue engineering and regenerative medicine approaches.

#### 3.2.1. Controlled Delivery of Nonviral Vectors

Supramolecular hydrogels based on block copolymer composed of poly(l-lysine) (PLL) segments for complexation of plasmid DNA encoding for the green fluorescent protein (GFP) and Pluronic^®^ F68 (PF68-PLL) to form inclusion complexes α-CD were produced [9] (Table 1). Both gelation time and mechanical strength could be modulated by tuning the amounts of F-68-PLL and α-CD. Likewise, the systems released DNA complexes for 3 days allowing for sustained transgene expression in a fibroblast cell line with reduced cytotoxicity.

Li et al. prepared triblock copolymers of methoxy-poly(ethylene glycol)-*b*-poly(ε-caprolactone)-*b*-poly[2-(dimethylamino)ethyl methacrylate] (MPEG-PCL-PDMAEMA) with well-defined cationic block lengths to condense pDNA in polyplexes. Of note, MPEG imparted stability to the pDNA polyplexes and also served as an anchoring segment when the pDNA polyplexes were encapsulated in α-CD-based supramolecular polypseudorotaxane hydrogels. In addition, the systems released pDNA in a sustained way for up to 6 days, leading to transfection levels comparable to those achieved with freshly prepared poly(ethylene imine) (PEI) polyplexes [13].

Supramolecular based CD hydrogels have also been manipulated as scaffolds for matrix-mediated gene transfection. By these lines, hydrogen bonding strengthened hydrogels were prepared by radical copolymerization of PEG methacrylated β-CD (PEG-β-CD) and 2-vinyl-4,6- diamino-1,3,5-triazine (VDT) monomer [39]. Immobilization of plasmid DNA onto the surface of hydrogels was achieved by hydrogen bonding between the base pairs and diaminotriazine, resulting in an efficient reverse gene transfection of the luciferase gene in a kidney cell line (COS-7) cultured on the gel surface.

CD-based polypseudorotaxane hydrogels have also been described as potential candidates to design injectable hydrogels for cancer therapy by providing a long term, sustained at tumor sites treatment while minimizing the exposition of non-target tissues [15,37].

Injectable supramolecular hydrogel systems were formed by complexations between α-CD and cationic MPEG-PCL-PEI copolymer. The resulting hydrogels were capable of forming polyplexes with reporter plasmid DNA and of providing a sustained release of pDNA in the form of polyplexes for up to 7 days. Of note, the incorporation of the antipoptotic Bcl-2 conversion gene in the systems resulted in an effective inhibition of tumor growth after 7 days in vivo when injecting into a solid tumor of nude mice [15]. More recently, the same authors involved a similar system incorporating a folic acid targeted group (MPEG-PCL-PEI-FA/α-CD) to co-deliver the chemotherapeutic paclitaxel and the antipoptotic Bcl-2 conversion gene in a tumor of mice [40]. Rapid solidification of the systems was noted after administration *via* peritumoral injection. Likewise, a significant prevention of the in vivo growth of therapeutic-resistant H460/Bcl-2 tumor was observed as a consequence of the sustained release of supramolecular hydrogel and targeting ability mediated by FA ligand.

A similar tendency was observed upon encapsulation of an MMP-9 shRNA plasmid (pMMP-9) into α-CD and PEGylated arginine-functionalized PLL dendron hydrogels [37].

#### 3.2.2. Controlled Delivery of Viral Vectors

While less explored than their nonviral vector counterparts, most of the attempts to design controlled delivery systems of viral vectors focused on the design of microcapsules of biodegradable polymers for the sustained release of the vector at the target place, and polymer conjugates that provide stealth, cell-targeted shells to the viral vectors [41,42,43]. In this regard, although viral gene transfer is highly efficient, its outcome can still be precluded by some barriers, such as high immunogenicity and inhibition of transduction in the presence of specific anticoagulants [31,32,33].

While recombinant adeno-associated virus (rAAV) vectors are considered the safest vectors for viral gene transfer, their translational use in patients might be impeded by the presence of neutralizing antibodies against the AAV capsid proteins in the host [32], especially by those present in the synovial fluid of patients with joint diseases [44]. Controlled delivery of rAAV vectors *via* polymeric biomaterials has already shown to be a potent way to overcome these issues [33]. Additionally, syringeable hydrogels that can be precisely placed in a specific site of the body using minimally invasive ways and that transform into depots for sustained release of active substances avoiding their diffusion to non-target places, have been largely pursued for different regenerative medicine approaches. Therefore, we recently generated syringeable polypseudorotaxane gels to produce materials that can durably deliver rAAV vectors for applications in cartilage regeneration [11] (Table 2). To achieve this goal, dispersions of Pluronic^®^ F68 (PF68) or Tetronic^®^ 908 (T908) containing either hyaluronic acid (HA) or chondroitin sulfate (CS) were prepared in PBS. α-CD was next added to form polypseudorotaxane gels. Compared with free vectors, the gels allowed to promote higher levels of transgene expression. CS (or HA)/PF68/α-CD gels rapidly released rAAV vectors while CS (or HA)/T908/α-CD gels provided sustained release, probably due to different interactions with the viral vectors. Incorporation of α-CD into CS (or HA)/PF68 gels resulted in higher rAAV concentrations and sustained levels of transgene expression in monolayer cultures of primary human bone marrow-derived mesenchymal stem cells (hMSCs) over time. In addition, HA increased both bioactivity and cytocompatibility of the gels [11].

**Figure 2:**
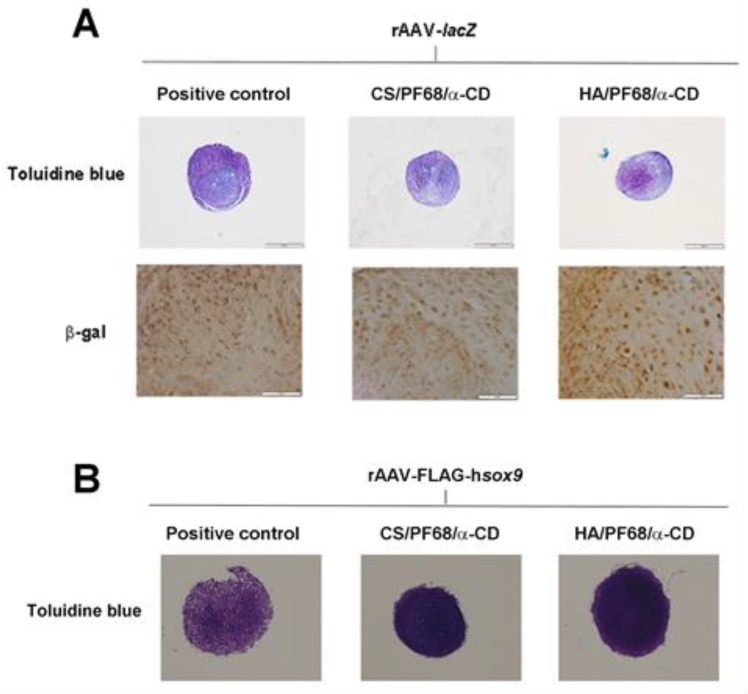
Controlled delivery of rAAV-*lacZ* (**A**) or rAAV-FLAG-h*sox9* (**B**) *via* polypseudorataxane hydrogels. Pellets were cultured in the absence (positive control; free rAAV form) or presence of rAAV-loaded hydrogels systems (Pluronic^®^ F68 (PF68)/ chondroitin sulfate (CS)/alpha-cyclodextrins (α-CD) or PF68/ hyaluronic acid (HA)/α-CD) for 21 days and monitored for chondrogenic differentiation (**A**,**B**: Toluidine blue staining: magnification ×4, all representative data) and β-galactosidase activity (**A**: β-gal immunounoreactivity: magnification ×20, all representative data).

Likewise, to study the potential of the systems for cartilage regeneration approaches, hydrogels were cultured for 21 days upon contact with hMSCs in a 3D aggregate culture model. Of note, no deleterious effects from the hydrogels were noted on the chondrogenic potential of the cells exhibiting no differences with those cells cultured in the absence of polyseudorotaxane systems (Figure 2A). Noteworthy, controlled delivery of a reporter gene (rAAV-*lacZ*) *via* HA/PF68/α-CD hydrogels resulted in the most effective gene transfer (Figure 2A). Similarly, superior chondrogenic differentiation was noted by delivery of the chondrogenic factor SOX9 (rAAV-FLAG-h*sox9*) *via* these hydrogel systems (Figure 2B). These systems might also be envisioned for the delivery of other morphogens capable of stimulating MSC differentiation to another lineages, such as osteoblasts.

## 4. Conclusions

Over the past decades, CD-based supramolecular hydrogels raised growing interest as biomaterials for drug and gene delivery approaches. Because of their unique properties of thixotropicity, biosafety, and easy modification, CD-based polypseudorotaxane hydrogels can be used as promising injectable delivery systems for controlled gene delivery. Likewise, the dilution process of these systems in contact with body fluids may be modulated by adjusting injection times or changing the molecular weight of the polymeric backbone [38].

Different CD-based polypseudorotaxane hydrogels have been produced to design systems able to provide a long term local availability of DNA vectors at a specific location and to stimulate several physiological mechanisms able to enhance the repair of injured tissues.

## Figures and Tables

**Table 1:** Controlled delivery of nonviral vectors *via* supramolecular-based CD hydrogels.

Polymers/CDs	Vectors	Outcomes	Approaches	Targets	References
PF68-PLL/α-CD	pDNA-GFP	Sustained pDNA delivery for 80 h; transfection efficiency ~14%	mouse fibroblast cells 3T3	n.s.	[9]
MPEG-PCL-PDMAEMA/α-CD	pDNA-*luc*	Sustained release of pDNA up to 6 days; transfection efficiency comparable to freshly prepared PEI polyplexes	COS-7 cells	n.s.	[13]
PEG-α-CD-cross-linked PVDT	pDNA-*luc*	Efficient reverse gene transfection of cells cultured on the gel surface	COS-7 cells	n.s.	[39]
MPEG-PCL-PEI/α-CD MPEG-PCL-PEIFA/α-CD	pDNA-GFP pDNA-Nur77	Sustained release of pDNA for 7 days; transfection efficiency of 63% at optimal weight ratio of 1.5; significant inhibition of therapeutic resistant tumor growth with high expression of Bcl-2 proteins Higher efficiency when combining the chemotherapeutic agent paclitaxel and the targeting ability of FA	HEK293 cells, tumor model (BALB/c nude mice)	tumor	[15,40]
MPEG-PLLD-Arg/α-CD	pMMP-9	Controlled release for 6 days; transfection efficiency up to 72%; sustained tumor growth inhibition after 21 days with good biocompatibility	HNE-1 cells, nude mice bearing HNE-1 tumors	tumor	[37]

Abbreviations: CD: cyclodextrin; Pluronic^®^ F68; PLL: poly(l-lysine); α-CD: alpha-CD; MPEG-PCL-PDMAEM:. methoxy-poly(ethylene glycol)-*b*-poly(ε-caprolactone)-*b*-poly[2-(dimethylamino)ethyl methacrylate]; PEG: poly-ethylene glycol; β-CD: beta-CD; PVDT: poly-2-vinyl-4,6-diamino-1,3,5-triazine; PEI: poly(ethylene imine); PLLD-Arginine-functionalized PLL dendron; pDNA: plasmid DNA; GFP: green fluorescent protein; *luc*: luciferase; Nur 77: Bcl-2 (B-cell lymphoma 2) conversion Nur77 gene; FA: folic acid; MMP-9: matrix metalloproteinase 9; HNE-1: human nasopharyngeal carcinoma HNE-1 cells; n.s.: not specified.

**Table 2:** Controlled delivery of viral vectors *via* supramolecular-based cyclodextrins (CD) hydrogels.

Polymers/CDs	Vectors	Outcomes	Approaches	Targets	References
CS (or HA)/PF68/α-CD CS (or HA)/T908/α-CD	rAAV-*lacZ*	Sustained release for 21 days; CS (or HA)/PF68/α-CD gels resulted in the highest rAAV concentrations and sustained levels of transgene expression over time	hMSCs	cartilage repair	[11]

Abbreviations: CD: cyclodextrin; CS: chondroitin sulfate; HA: hyaluronic acid; PF68: Pluronic^®^ F68; T908: Tetronic^®^ 908; α-CD: alpha-CD; hMSCs: human bone marrow-derived mesenchymal stem cells.
